# A nomogram model based on pre-treatment and post-treatment MR imaging radiomics signatures: application to predict progression-free survival for nasopharyngeal carcinoma

**DOI:** 10.1186/s13014-023-02257-w

**Published:** 2023-04-11

**Authors:** Mi-Xue Sun, Meng-Jing Zhao, Li-Hao Zhao, Hao-Ran Jiang, Yu-Xia Duan, Gang Li

**Affiliations:** 1 Department of Radiation Oncology, The First Affiliated Hospital of Wenzhou Medical University, Wenzhou, Zhejiang People’s Republic of China; 2 Department of Radiology, The First Affiliated Hospital of Wenzhou Medical University, Wenzhou, Zhejiang People’s Republic of China

**Keywords:** Nasopharyngeal carcinoma, Magnetic resonance imaging, Radiomics, Nomogram, Progression-free survival, Prognosis

## Abstract

**Background:**

To establish a novel model using radiomics analysis of pre-treatment and post-treatment magnetic resonance (MR) images for prediction of progression-free survival in the patients with stage II–IVA nasopharyngeal carcinoma (NPC) in South China.

**Methods:**

One hundred and twenty NPC patients who underwent chemoradiotherapy were enrolled (80 in the training cohort and 40 in the validation cohort). Acquiring data and screening features were performed successively. Totally 1133 radiomics features were extracted from the T2-weight images before and after treatment. Least absolute shrinkage and selection operator regression, recursive feature elimination algorithm, random forest, and minimum-redundancy maximum-relevancy (mRMR) method were used for feature selection. Nomogram discrimination and calibration were evaluated. Harrell’s concordance index (C-index) and receiver operating characteristic (ROC) analyses were applied to appraise the prognostic performance of nomograms. Survival curves were plotted using Kaplan–Meier method.

**Results:**

Integrating independent clinical predictors with pre-treatment and post-treatment radiomics signatures which were calculated in conformity with radiomics features, we established a clinical-and-radiomics nomogram by multivariable Cox regression. Nomogram consisting of 14 pre-treatment and 7 post-treatment selected features has been proved to yield a reliable predictive performance in both training and validation groups. The C-index of clinical-and-radiomics nomogram was 0.953 (all *P* < 0.05), which was higher than that of clinical (0.861) or radiomics nomograms alone (based on pre-treatment statistics: 0.942; based on post-treatment statistics: 0.944). Moreover, we received Rad-score of pre-treatment named RS1 and post-treatment named RS2 and all were used as independent predictors to divide patients into high-risk and low-risk groups. Kaplan–Meier analysis showed that lower RS1 (less than cutoff value, − 1.488) and RS2 (less than cutoff value, − 0.180) were easier to avoid disease progression (all *P* < 0.01). It showed clinical benefit with decision curve analysis.

**Conclusions:**

MR-based radiomics measured the burden on primary tumor before treatment and the tumor regression after chemoradiotherapy, and was used to build a model to predict progression-free survival (PFS) in the stage II–IVA NPC patients. It can also help to distinguish high-risk patients from low-risk patients, thus guiding personalized treatment decisions effectively.

**Supplementary Information:**

The online version contains supplementary material available at 10.1186/s13014-023-02257-w.

## Background

Nasopharyngeal carcinoma (NPC) is a rare malignant tumor of head and neck worldwide [1], but its incidence is high in South China. Worldwide, there are about 133,000 new NPC cases in 2020, accounting for 0.7% of all cancers diagnosed in that year [2]. Intensity modulated radiation therapy (IMRT) is the standard radiotherapy of NPC at present [3, 4]. Although the introduction of IMRT can control local metastasis and improve prognosis, the mortality rate of NPC is still high. Likewise, the incidence of progress remains high. Previous research has shown that about 10–15% of patients had locoregional recurrence and distant metastasis events in the first 2 years after treatment, and only 72.9% of patients had no disease progression within 2 years [5]. It is indispensable to identify patients with a high risk of recurrence by some means and give certain individualized intervention as soon as possible.

In the past decade, medical imaging has made rapid development [6, 7]. Meanwhile, due to the emergence of radiomics, it has transformed traditional qualitative science to quantitative science, greatly broadening the application scenarios of conventional medical imaging in clinical oncology. This method of converting acquired image data into quantitative descriptors of tumor histological characteristics enables clinicians to enhance image interpretation capabilities. As an improvement of methodology, radiomics is a process of mining in data’s essence [8, 9]. Encrypted digital medical images can be converted into multi-dimensional quantitative data. Our integration of these data makes it possible to attach the prediction of various clinical outcomes.

As one of the most common tools for risk stratification, however, tumor-node-metastasis (TNM) staging system shows poor prognostic performance in NPC patients [10]. Patients with nasopharyngeal carcinoma in the same stage often show different clinical outcomes and prognosis [11, 12]. Patients with the same TNM stage and similar treatments often show different survival outcomes, and the difference of long-term survival rates is significant in each stratification [13]. All these are attributed to the fact that TNM staging system is based on anatomy without regards to intrinsic biological heterogeneity [14, 15]. Besides, other risk factors are ignored, which lead to its obvious limitations. For this reason, there is an attempt to develop a novel model that can better predict the prognosis of NPC patients.

## Materials and methods

### Patient selection

Between March 2017 and December 2019, a total of 120 patients diagnosed with stage II–IVA NPC and treated with IMRT and chemotherapy in the First Affiliated Hospital of Wenzhou Medical University were recruited in our cohorts.

The specific eligibility criteria were: (1) patients with biopsy-proven primary nasopharyngeal carcinoma; (2) patients who received complete chemoradiotherapy; (3) patients who underwent the same 3.0-T magnetic resonance scanner to obtain imaging data 3 weeks before treatment start and 3 weeks after treatment end, including at least T2-weight images (T2WI); (4) patients with availability and integrity of clinical and treatment data. The exclusion criteria were: (1) patients with history of anticancer therapy before baseline magnetic resonance imaging (MRI) scans or incomplete radiotherapy and chemotherapy; (2) patients who lacked of T2WI or contrast-enhanced T1-weighted image (CE-T1WI) data; (3) patients with poor quality MRI data which was difficult to draw or measure; (4) patients with serious heart, lung, liver and kidney diseases; (5) patients with other parts of body primary malignant tumors.

On the basis of these criteria, a total of 120 patients were enrolled in our study. Patients were randomly allocated to the training (n = 80) and validation group (n = 40) at a 2:1 ratio via computer software-generated random numbers.

The retrospective study was conducted in accordance with the Helsinki declaration. Since this study was based on routine MRI examination and analysis of clinical data, individual informed consent was not required. Each patient was evaluated by an experienced pathologist and two independent radiologic oncologists based on pathology and imaging findings. Tumor staging was performed in consensus according to the 8th Edition of American Joint Committee on Cancer/Union for International Cancer Control (AJCC/UICC) TNM Staging System.

### Treatment

Treatment regimen for all patients followed the clinical practice guidelines of National Comprehensive Cancer Network (NCCN) for Head and Neck (H&N) cancers. Treatment of NPC patients included local radiotherapy and systemic chemotherapy. According to guidelines, patients (stage II–IVA) received neoadjuvant or adjuvant chemotherapy and/or concurrent chemotherapy with radiotherapy. Our chemotherapy consisted of every 3-week platin-based regimens for 4–6 cycles. During the research, all patients received IMRT, once a day for five days a week with the cumulative radiation doses ranged from 66 to 76 Gy (average, 70 Gy) for the primary tumor and involved lymph nodes [16]. Besides, radiation dose to the high-risk drainage area was 60 Gy, and that to the low-risk preventive radiation area was 54 Gy.

### Follow-up and clinical endpoint

After treatment, all patients were followed up every 3–6 months in the first 3 years, and then every 6–12 months until death, focusing on the re-examination of nasopharyngeal and cervical contrast-enhanced magnetic resonance (MR). Patients were asked to have an imaging examination of chest and abdomen once a year. If any suspicious signs of recurrence or progression appeared, additional examinations were required.

In our study, following baseline data were collected from each patient, including medical history and physical examination (age, gender, smoking status, alcohol consumption), histopathological information (histological grade, pathological subtype), routine blood chemical analysis (white blood cell (WBC), platelet (PLT), hemoglobin (HGB), neutrophil (NEUT) counts, levels of plasma carcinoembryonic antigen (CEA), imaging and endoscopic examinations, and stage (T-stage, N-stage and overall stage) within 2 weeks prior to treatment. These data were obtained from the records in medical record system.

Patient follow-up was calculated from the date of pathological diagnosis to the date of the last outpatient examination or death. If patient's recent visit was not recorded in medical record, we would try to get in touch by telephone. All follow-ups ended in December 2021. The shortest follow-up time was 12.4 months. The longest follow-up time in our collection cases was 53.6 months and major patients have not reached the endpoint yet, progression-free survival (PFS) performed well as the final evaluation parameter compared to overall survival. In order to carry out individualized treatment earlier and avoid long-term follow-up, period combined death, locoregional recurrence and distant metastasis, was selected as survival destination ultimately. PFS referred to the time from the end date of therapy to recurrence of any part or death from any reason, whichever occurred first.

The diagnosis of all local recurrence was confirmed by nasopharyngoscopy and biopsy and/or imaging evidence. Regional recurrence was detected by clinical examination of neck. In suspicious cases, fine needle aspiration biopsy or MRI scans would be used. Besides, the diagnosis of distant metastases was based on clinical symptoms, physical examinations and imaging examinations including chest radiography, ultrasound, bone scan, computed tomography (CT), MRI and positron emission tomography (PET)/CT.

### MRI data acquisition

Images conforming to standards of the Digital Imaging and Communications in Medicine (DICOM) were obtained before and after radiotherapy from the records of Picture Archiving and Communication System (PACS) of the First Affiliated Hospital of Wenzhou Medical University. All images were generated after scanning by a Signa HDX 3.0-T MR scanner (General Electric Medical Systems). It was equipped with a 8-channel head–neck phased array coil and be used to provide required signal sequences, including T1-weight images, T2WI, CE-T1WI, etc. In the axial T2-weighted images, parameters were set as followed: repetition time (TR) = 4080 or 4100 ms, echo time (TE) = 85 ms, field of view (FOV) = 220 × 220 or 240 × 240 mm, matrix (Mat) = 320 × 192, fip angle (FA) = 90°, number of excitations (NEX) = 2.0, slice thickness = 4.0 mm, slice spacing = 1.0 mm, and number of scanning slices = 20.

All patients underwent 3.0-T MRIs within 3 weeks before and after treatment. MRI scans usually contain sequences of unenhanced T1- and T2-weighted, contrast-enhanced T1-weighted and fat-suppressed T1-weighted. Outline, intensity, and gray scale of nasopharyngeal tumors are obviously different from normal tissues on T2WI and CE-T1WI sequences. However, enhanced magnetic resonance imaging sequences are not available for every patient. Besides, scanning cannot be attached at all treatment time points, which results in unavailability in several cases. Consequently, the baseline MRI data we used were chosen to be drawn on T2WI sequences. At last, axial T2-weighted DICOM images were attached in Institution’s PACS for feature selection and did not need any preprocessing or standardization to original images.

### Tumor segmentation

All feature data were extracted and generated from the regions of interest (ROIs), so its selection and delineation were extremely important and challenging. Multiparameter MRIs were performed with a 3.0-T MR scanner on each patient, including T2-w (axial) images and contrast-enhanced T1-w (axial, coronal and sagittal) images. The forementioned images were extracted from the PACS and loaded into a 3D Slicer for manual segmentation (open-source software: version 4.11; https://www.slicer.org/). The axial T2-w images were depicted on each layer and the contrast-enhanced T1-w images in axial, coronal and sagittal positions were used to guide segmentation for ROIs. All images were segmented by a radiologic oncologist and an experienced radiologist. Furthermore, each segmentation was also validated and evaluated by a senior radiologist engaging in the field of head and neck cancer. Any problems were resolved by consulting another senior radiologist.

### Radiomics feature extraction

**Fig. 1:**
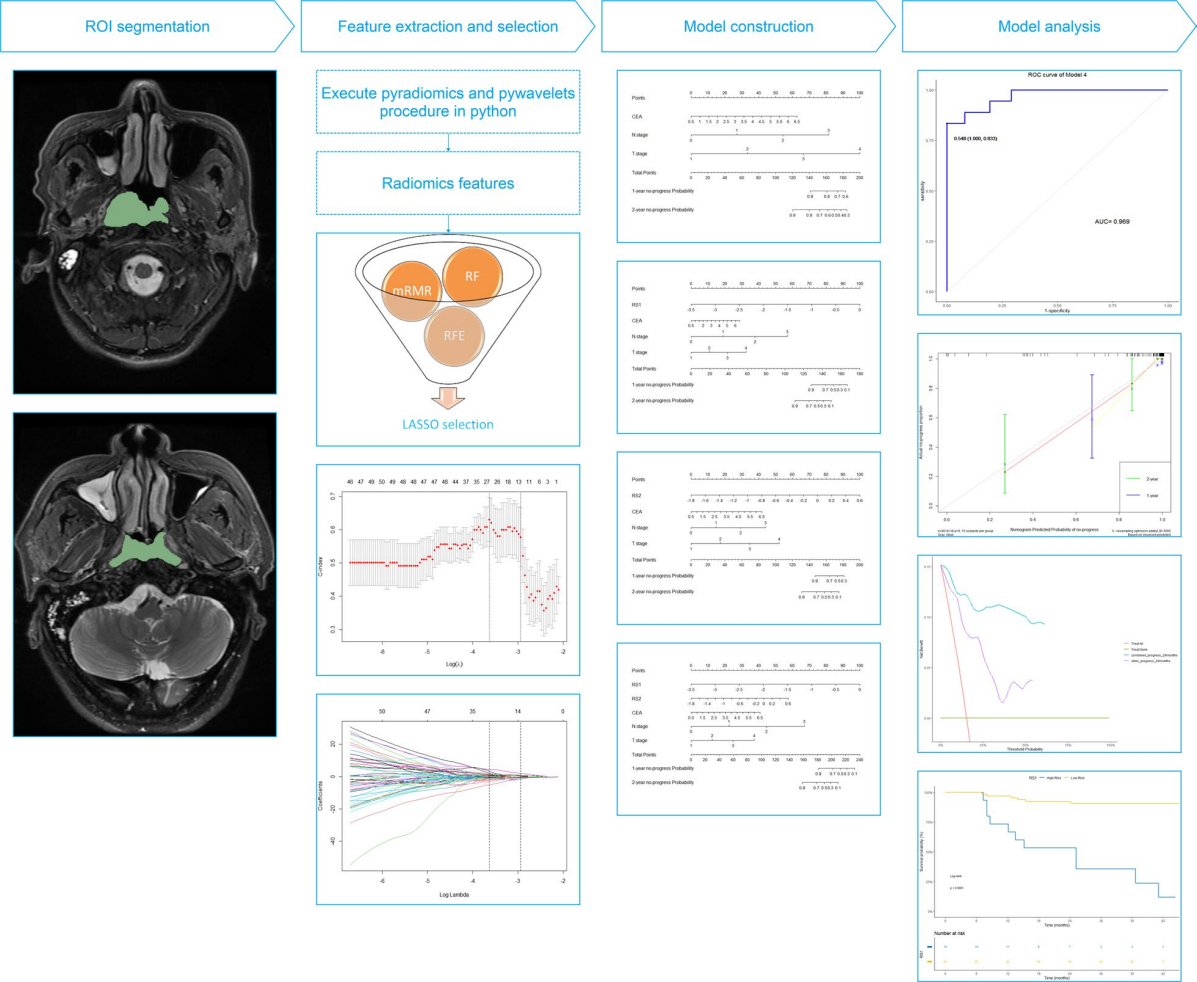
Radiomics workflow in this study

Radiomics extracts high-resolution images from ROIs in the target and it can effectively transform medical images into multi-dimensional minable features by quantifying information on tumor shape, size, volume, as well as texture, intensity features (Fig. 1). Imaging feature extraction methods utilize the PyRadiomics platform (https://pypi.org/project/pyradiomics/) to achieve on Anaconda3 64 bit software (https://www.anaconda.com/). Relying on a large number of feature algorithms on this platform, it is used to extract standardized quantitative imaging features. In our research, one thousand one hundred and thirty-three features were extracted and then divided into three classes as follows: shape-based features, first-order statistics, and texture features [including gray level cooccurrence matrix (GLCM), gray level run length matrix (GLRLM), gray level size zone matrix (GLSZM), gray level dependence matrix (GLDM), and neighbouring gray tone difference matrix (NGTDM)] (https://pyradiomics.readthedocs.io/en/latest/features.html).

We directly used DICOM images generated from scanner. And then, outlining regions of interest and the part where ROI located in was used to calculate corresponding radiation features. All the data, containing radiomics features and clinical measures, were normalized by converting the data into new scores with an average of 0 and a standard deviation of 1 (z-score) in the above procedures.

### Statistical analysis

For clinical risk factors, the collected data were non-normal distribution parameters. Firstly, univariate analysis was applied to test individual effect of each variable on PFS in primary cohort. The associations between PFS and T-stage, N-stage, total stage, age, gender and other factors were analyzed by Cox proportional hazard regression model afterwards. The variables that reached significant level in the univariate analysis (*P* < 0.1) were included in the multivariate analysis. Then, multivariate Cox regression analysis was performed to determine independent prognostic factors and those with *P* < 0.05 were included in our clinical model.

## Results

### Clinical characteristics

The appendix summarized the baseline clinicopathological features of 120 NPC patients, including 28 cases of recurrence (10 locoregional relapses and 18 distant metastases). The average PFS was 17.2 months (range, 7.0–46.1 months). There was no tumor recurrence in 92 patients, while the average PFS was 33.7 months (range, 7.4–45.6 months). The minimum follow-up time to determine progression-free survival was 12.4 months from the date of diagnosis, while the maximum follow-up time was 53.6 months (median, 37.9 months).

Besides, Wilcoxon–Mann–Whitney test was applied to analyze the significance of individual variables for non-normal distribution parameters. Differences between the training and validation cohorts in terms of age, gender, smoking status, alcohol consumption, pathologic type, T-stage, N-stage, overall stage, WBC, NEUT, PLT, HGB, CEA, PFS, follow-up time, survival status, or progress were assessed by Mann–Whitney U test. The results showed that no significant differences were found between them (*P* = 0.242–0.990) and all were reported in groups with average value and standard deviation. The details of statistic are described in Table 1.

**Table 1:** Characteristics of Patients with NPC in the Training and Validation Cohorts

Characteristic	Entire cohort	Training cohort	Validation cohort	*P*-value
	(n = 120)	(n = 80)	(n = 40)	
Age (years)				0.898
Mean (SD)	54.5 (12.6)	54.8 (12.8)	53.7 (12.4)	
Median [Min, Max]	55 [23, 81]	55 [23, 81]	55 [25, 76]	
Gender				0.456
Male	94 (78.3%)	60 (75.0%)	34 (85.0%)	
Female	26 (21.7%)	20 (25.0%)	6 (15.0%)	
Smoking				0.921
No	78 (65.0%)	51 (63.8%)	27 (67.5%)	
Yes	42 (35.0%)	29 (36.3%)	13 (32.5%)	
Drinking				0.700
No	84 (70.0%)	54 (67.5%)	30 (75.0%)	
Yes	36 (30.0%)	26 (32.5%)	10 (25.0%)	
WHO pathologic typea				0.861
Type I	3 (2.5%)	2 (2.5%)	1 (2.5%)	
Type II	49 (40.8%)	34 (42.5%)	15 (37.5%)	
Type III	68 (56.7%)	44 (55.0%)	24 (60.0%)	
Pre-treatment T stage				0.984
T1	23 (19.2%)	14 (17.5%)	9 (22.5%)	
T2	26 (21.7%)	19 (23.8%)	7 (17.5%)	
T3	57 (47.5%)	37 (46.3%)	20 (50.0%)	
T4a	14 (11.7%)	10 (12.5%)	4 (10.0%)	
Pre-treatment N stage				0.879
N0	13 (10.8%)	10 (12.5%)	3 (7.5%)	
N1	31 (25.8%)	18 (22.5%)	13 (32.5%)	
N2	70 (58.3%)	47 (58.8%)	23 (57.5%)	
N3	6 (5.0%)	5 (6.3%)	1 (2.5%)	
Overall stage				0.988
II	19 (15.8%)	12 (15.0%)	7 (17.5%)	
III	80 (66.7%)	53 (66.3%)	27 (67.5%)	
IVa	21 (17.5%)	15 (18.8%)	6 (15.0%)	
Pre-treatment WBC (10^12/L)				0.375
Mean (SD)	6.64 (2.11)	6.45 (2.07)	7.03 (2.16)	
Median [Min, Max]	6.12 [2.23, 12.8]	5.99 [2.23, 12.8]	6.62 [2.84, 11.9]	
Pre-treatment NEUT (10^9/L)				0.265
Mean (SD)	4.24 (1.80)	4.05 (1.74)	4.62 (1.88)	
Median [Min, Max]	3.77 [0.70, 9.66]	3.48 [0.98, 9.44]	4.01 [0.70, 9.66]	
Pre-treatment PLT (10^9/L)				0.853
Mean (SD)	246 (90.8)	250 (99.4)	240 (71.1)	
Median [Min, Max]	236 [62, 724]	235 [62, 724]	240 [90, 459]	
Pre-treatment HGB (g/L)				0.337
Mean (SD)	141 (14.7)	139 (14.8)	143 (14.1)	
Median [Min, Max]	142 [96, 182]	139 [96, 182]	144 [114, 170]	
Pre-treatment CEA (ug/L)				0.242
Mean (SD)	2.29 (1.41)	2.13 (1.29)	2.59 (1.61)	
Median [Min, Max]	1.9 [0.5, 7.2]	1.8 [0.5, 6.2]	2.1 [0.8, 7.2]	
Progression-free survival (months)				0.944
Mean (SD)	29.9 (11.0)	30.1 (11.0)	29.4 (11.2)	
Median [Min, Max]	30.7 [7.0, 46.1]	32.6 [7.0, 46.1]	30.4 [7.0, 43.9]	
Follow-up time (months)				0.889
Mean (SD)	37.1 (8.92)	37.4 (9.19)	36.5 (8.43)	
Median [Min, Max]	37.9 [12.4, 53.6]	38.4 [12.4, 53.6]	37.4 [18.1, 48.4]	
Survival status				0.954
No	92 (76.7%)	62 (77.5%)	30 (75.0%)	
Yes	28 (23.3%)	18 (22.5%)	10 (25.0%)	
Progress				0.823
No	92 (76.7%)	62 (77.5%)	30 (75.0%)	
Distant metastases	18 (15.0%)	13 (16.3%)	5 (12.5%)	
Locoregional relapses	10 (8.3%)	5 (6.3%)	5 (12.5%)	

A Cox regression model based on clinical variables was established to learn the importance of each variable. Finally, T-stage, N-stage, and CEA were selected as independent risk factors to be included in subsequent clinical prediction model (Table 2).

**Table 2:** The results of univariate and multivariate cox proportional hazard analysis in this study

Variable	Univariate cox regression		Multivariate cox regression	
	*P*-value	Hazard ratio (95% confidence interval)	*P*-value	Hazard ratio (95% confidence interval)
Age	0.968	0.96 (0.11–8.62)	–	–
WBC	0.289	3.34 (0.36–31.03)	–	–
NEUT	0.187	4.27 (0.49–36.86)	–	–
PLT	0.509	2.59 (0.15–43.65)	–	–
HGB	0.586	2.14 (0.14–32.82)	–	–
CEA	0.013	12.64 (1.72–92.68)	0.0196	20.49 (1.62–258.62)
*Gender*				
Male				
Female	0.782	1.16 (0.41–3.30)	–	–
*Smoking*				
No				
Yes	0.523	1.37 (0.52–3.61)	–	–
*Drinking*				
No				
Yes	0.475	0.66 (0.22–2.04)	–	–
*WHO pathologic typea*				
Type I				
Type II	0.769	0.74 (0.09–5.73)	–	–
Type III	0.181	0.23 (0.03–1.98)	–	–
*T stage*				
T1				
T2	0.841	0.75 (0.05–12.05)	0.7037	1.78 (0.09–34.18)
T3	0.167	4.34 (0.54–34.77)	0.0089	44.81 (2.59–774.89)
T4a	0.014	14.91 (1.72–128.92)	0.0007	253.43 (10.21–6287.79)
*N stage*				
N0				
N1	0.568	1.94 (0.20–18.69)	0.1164	8.17 (0.59–112.41)
N2	0.438	2.26 (0.29–17.65)	0.1435	5.63 (0.56–57.00)
N3	0.057	9.09 (0.94–88.07)	0.0004	352.82 (13.69–9094.31)
*Overall stage*				
II				
III	0.998	82,836,007.61	–	–
IVa	0.997	488,669,656.57	–	–

### Methodology of feature selection

Three feature selection methods with different principles were applied to screen the radiomics data. The first was using recursive feature elimination (RFE) algorithm to select features. It was a wrapping method which was implemented by Python software (version 3.9.12; https://www.python.org/). The RFE algorithm selected and fetched the best or worst features by building the model repeatedly (use the logistic regression model in this example). And only one feature with the smallest weight coefficient was deducted at a time in our experiment. We compared the regression coefficients as prediction effect scores, and then repeated the process over the remaining features until all features were fetched [15]. In this process, the order in which features were taken out was the importance order of features. In the present study, L2-norm as the penalty term was used in regularized logistic regression [17]. Regularization reduced over-fitting, improved the generalization capacity effectively, and made learner more stable and efficient by adding penalty terms to existing model. Among 1133 extracted features, the remaining number of selected features was set to 113, which was about one-tenth of the total number of samples. It was a suitable screening ratio based on previous experience.

The second method was random forest algorithm, that was used in combination with embedding method for feature selection. All process were carried out in Python. We calculated the contribution of each feature on each tree in the random forest, took the average of contributions, compared them between features, and sorted them in descending order, then determined features to be eliminated by setting an appropriate threshold. With the aim of picking out the top 10% features, threshold was set at nearly 0.0019 eventually.

Third, we used the minimum-redundancy maximum-relevancy (mRMR) criteria [18] to screen features, which was a filtering method scoring each feature according to the correlation. We calculated the mutual information (MI) between features and output variable. Out of the comparison of MI, we ranked features that were most relevant to the final output, but least redundant relevant to each other. And then, we set an appropriate threshold and ended up with 113 potential interesting features. All procedures performed in R (version 4.1.3; https://www.r-project.org/).

**Fig. 2:**
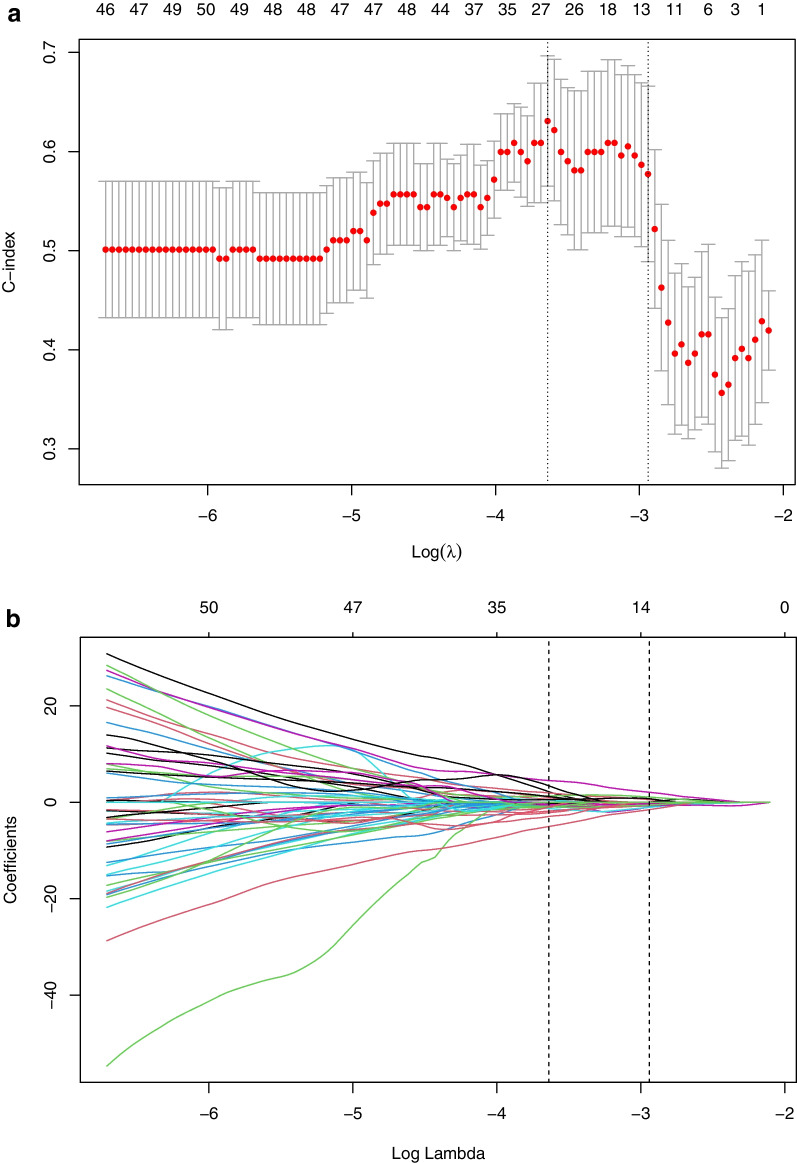
Selection of radiomics features before treatment via the least absolute shrinkage and selection operator (LASSO) Cox regression model. Tuning parameter (λ) selection in this model used tenfold cross-validation via the minimum criteria. **A** The Harrell’s concordance index (C-index) was plotted versus log(λ). Dotted vertical lines were drawn at the optimal values by using the minimum criteria and the 1 standard error of the minimum criteria (the 1-SE criteria), and the line on the right with λ value of 0.05284452 was chosen according to tenfold cross-validation. **B** LASSO coefficient profiles of the 278 radiomics features. A coefficient profile plot was generated versus value of log (λ). Two vertical lines were drawn at the value selected using tenfold cross-validation, where optimal λ pointed to 12 nonzero coefficients

**Fig. 3:**
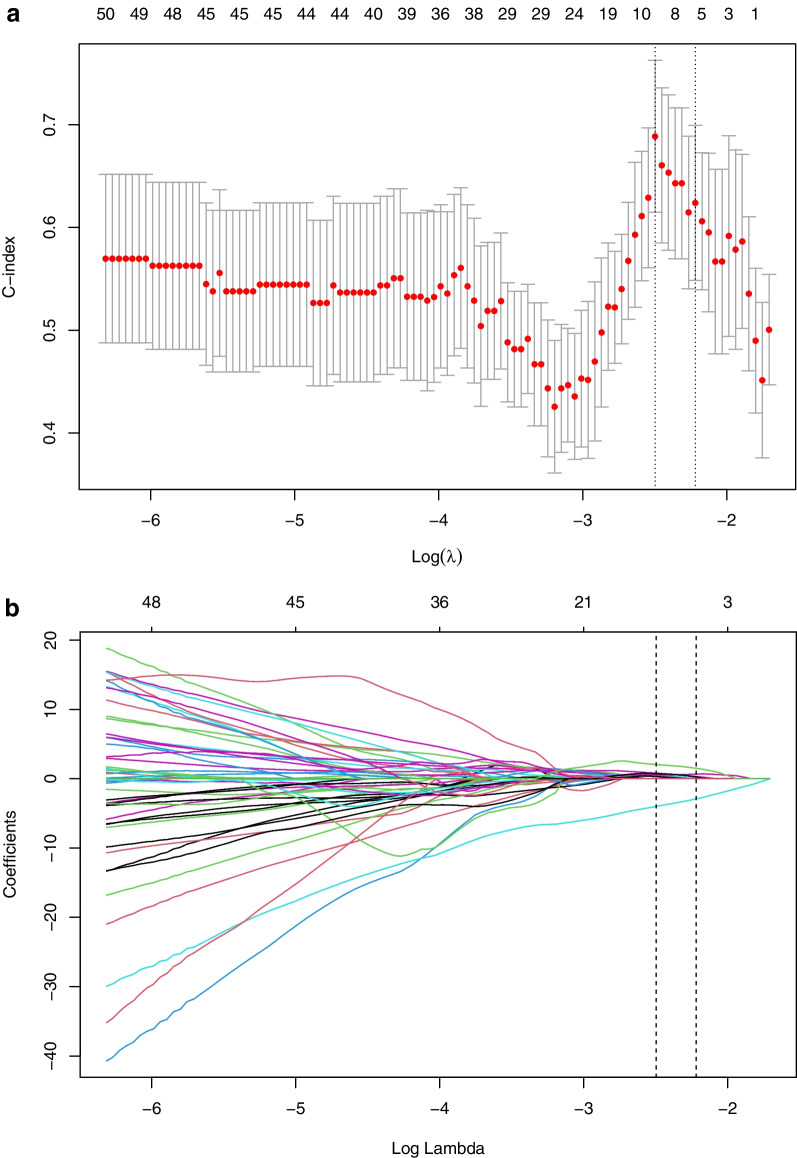
Selection of radiomics features after treatment via the LASSO Cox regression model. Tuning parameter (λ) selection in this model used tenfold cross-validation via the minimum criteria. **A** The C-index was plotted versus log(λ). Dotted vertical lines were drawn at the optimal values by using the minimum criteria and the 1 standard error of the minimum criteria (the 1-SE criteria), and the line on the right with λ value of 0.1087473 was chosen according to tenfold cross-validation. **B** LASSO coefficient profiles of the 299 radiomics features. A coefficient profile plot was generated versus value of log (λ). Two vertical lines were drawn at the value selected using tenfold cross-validation, where optimal λ pointed to 9 nonzero coefficients

Further merging the features obtained by the above three algorithms and selecting were performed using lasso regression analysis in the next step. Least absolute shrinkage and selection operator (LASSO), as a machine learning algorithm, is suitable for analyzing high-dimensional data with relatively small sample size. LASSO aims to avoid over-fitting [19], and its mechanism is to reduce the regression coefficients of many features to zero, remove less influential factors, diminish the difficulty of learning tasks, make the model easy to understand, and allow the identification of imaging features related to the results most significantly. The data with a much larger number of features than samples were produced by radiomics analysis. Consequently, the combination of LASSO and radiomics might achieve the purpose of selecting appropriate biomarkers. In LASSO implementation step, based on the maximum Harrell’s concordance index (C-index), we got 12 relatively valuable features from 278 texture features prior to treatment and 9 from 299 post-treatment features. Details of LASSO analysis are presented in Figs. 2, 3.

### Radiomics signature building

**Fig. 4:**
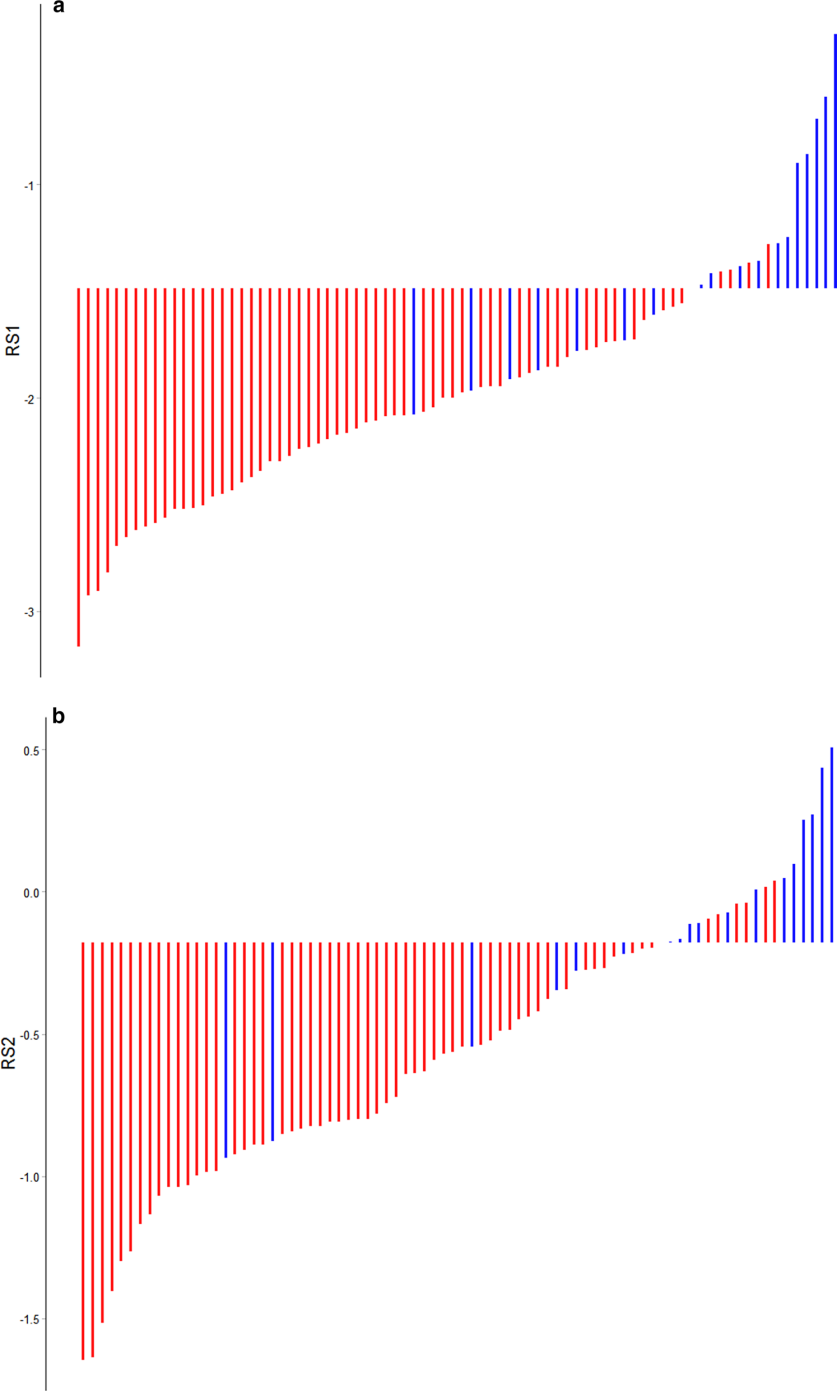
Distributions of Rad-score of disease progression and no-progression groups in training cohort: **A** RS1. **B** RS2. Red bars showed points for patients who survived without locoregional recurrence or distant metastasis, while blue bars showed points for those who experienced progression or died.

Multivariate regression analysis was applied to establish a clinical prediction model using the training cohort that included radiomics features and independent clinicopathological risk factors. Besides, considering the texture features might be interlocked and not independent from each other, we tried to introduce a radiomics signature that combined selected imaging features with non-zero coefficients. The product of each feature retained in the model and its corresponding regression coefficient (β) was summed to obtain radiomics score (Rad-score) [20–22]. We divided Rad-score into RS1 which originated from pre-treatment data and RS2 which originated from post-treatment data. The specific formulas are shown in Table 3 below. In addition, distributions of RS1 and RS2 of progression and no-progression groups for the training cohort are shown in Fig. 4.

**Table 3:** Two formulas concerning the Rad-score

	Radiomics features		Coefficients
*a. Formulas for calculating pre-treatment Rad-score (RS1)*			
RS1 =	original_shape_Elongation	*	− 0.74165845
+	original_shape_Flatness	*	− 0.19308606
+	original_glszm_LargeAreaHighGrayLevelEmphasis	*	− 0.96268135
+	log.sigma.3.mm.3D_firstorder_Skewness	*	− 1.52187110
+	log.sigma.3.mm.3D_glcm_InverseVariance	*	− 1.05523619
+	wavelet-HHH_glcm_MCC	*	− 0.65258808
+	wavelet-HHH_gldm_SmallDependenceLowGrayLevelEmphasis	*	0.03921352
+	wavelet-HHH_gldm_DependenceVariance	*	− 0.58868526
+	wavelet-HHH_glszm_SmallAreaLowGrayLevelEmphasis	*	0.75365003
+	wavelet-HLH_firstorder_Mean	*	0.53801631
+	wavelet-HLL_glcm_Autocorrelation	*	2.09368963
+	wavelet-LHH_firstorder_Skewness	*	1.09442341
+	wavelet-LHH_glcm_Imc1	*	− 0.58413612
+	wavelet-LLH_glcm_Idmn	*	− 0.32246341
*b. Formulas for calculating post-treatment Rad-score (RS2)*			
RS2 =	log.sigma.2.mm.3D_ngtdm_Contrast	*	0.33057259
+	log.sigma.3.mm.3D_glszm_SizeZoneNonUniformity	*	0.68298741
+	wavelet-HHH_firstorder_Median	*	− 2.91041062
+	wavelet-HLH_gldm_LargeDependenceHighGrayLevelEmphasis	*	0.07259247
+	wavelet-LLH_glcm_Autocorrelation	*	0.06522993
+	wavelet-LLL_glcm_ClusterProminence	*	0.25489697
+	wavelet-LLL_glcm_DifferenceVariance	*	1.49013870

### Multiple models establishing

We formed eight different models, include, (Model 1 and 5) model incorporating clinical response predictor (T stage, N stage, and CEA levels) in the construction of clinical nomogram, (Model 2 and 6) model containing clinical predictors and RS1, (Model 3 and 7) model containing clinical predictors and RS2, (Model 4 and 8) model constructed from RS1, RS2 and the aforementioned clinical factors. Data used in Model 1–4 were collected from training set. Likewise, data used in Model 5–8 were collected from testing set. The results of the models’ performances are described in Table 4.

**Table 4:** Performance of models

	Accuracy	Precision	Recall	F_measure	AUC	C-index
*Training cohort*						
Model 1	0.800	0.583	0.389	0.467	0.841	0.861
Model 2	0.950	0.938	0.833	0.882	0.955	0.942
Model 3	0.888	0.800	0.667	0.727	0.928	0.944
Model 4	0.963	1.000	0.833	0.909	0.969	0.953
*Validation cohort*						
Model 5	0.750	0.500	0.200	0.286	0.762	0.704
Model 6	0.800	0.625	0.500	0.556	0.883	0.841
Model 7	0.800	0.667	0.400	0.500	0.870	0.817
Model 8	0.900	0.800	0.800	0.800	0.923	0.877

**Fig. 5:**
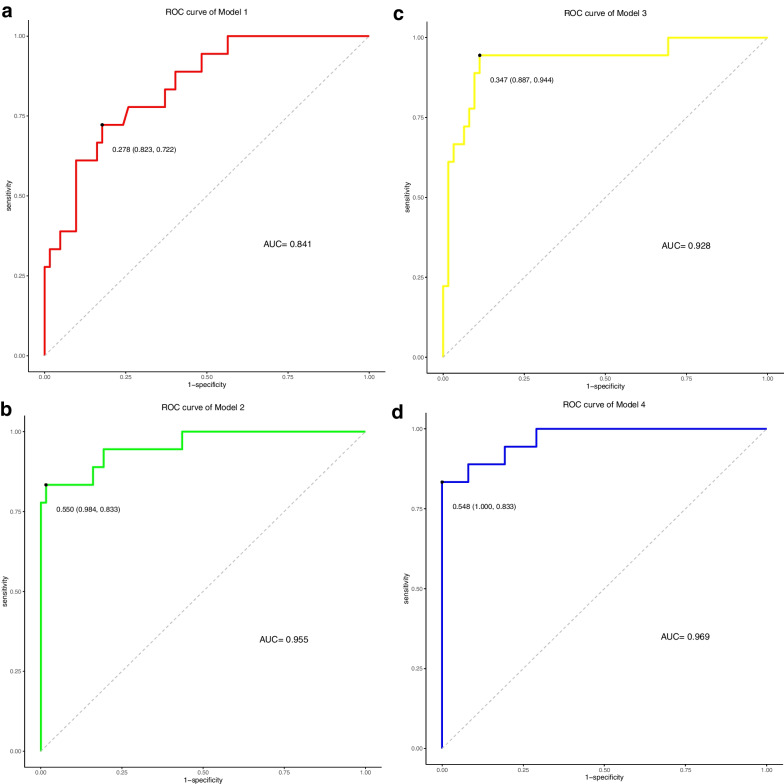

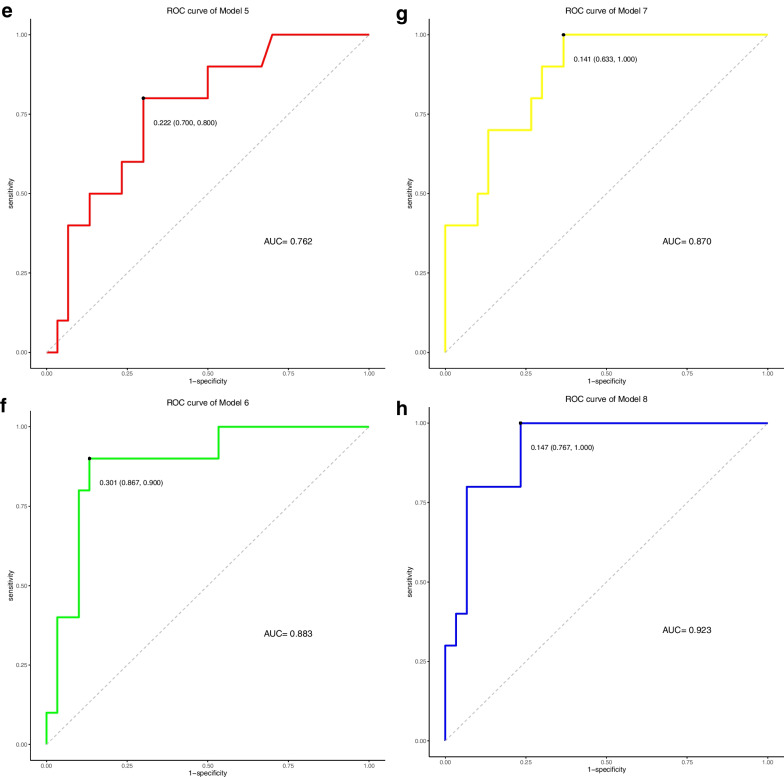
Receiver operating characteristic (ROC) curves for progression-free survival (PFS) models, which were based on data before and after treatment. **A**, **E** clinical variables, **B**,** F** clinical variables and RS1, **C**, **G** clinical variables and RS2, and **D**, **H** clinical variables integrated with RS1 and RS2 in the training and validation cohorts, respectively

We further evaluated the prediction efficiency of the above eight models by comparing the value of C-index. The conclusion was drawn that, the combination of double Rad-scores with clinical risk factors as composite clinical-and-radiomics models, whose receiver operating characteristic (ROC) curves were superior to clinical variables alone and clinical in conjunction with single pre-treatment or post-treatment radiomics signature in both the training and validation (Fig. 5). As for C-index, the composite clinical-and-radiomics nomogram predicted PFS better (0.953, 95% confidence interval (CI) 0.923–0.984) than clinical nomogram (0.861, 95%CI 0.796–0.926) or the single period radiomics models (0.942, 95%CI 0.904–0.980; 0.944, 95%CI 0.915–0.973) did (*P* < 0.01). A similar performance occurred in the validation set (C-index, 0.877 vs. 0.704 vs. 0.841 vs. 0.817) (*P* < 0.05), and the prediction performance of multiple models was good and consistent with our expectation. On the other hand, we have performed internal cross-validation (described in Additional file 1). The results are consistent with the previous ones. This method helps to improve the generalization performance, increase the credibility and stability of models, and minimize the sampling deviation.

### Creation and performance of an individualized radiomics nomogram

After selecting tumor texture features and clinical factors, a prognostic model, which took progression-free survival as endpoint, was established by using the data of training cohort and Cox regression. Using this model combined with selected radiomics and clinical features, a radiomics nomogram came into being. It performed incremental prognostic value of radiomics features in traditional staging system. Our nomogram provides clinicians with a quantitative tool to predict individualized no-progress survival, which can evaluate for discrimination and clinical utility subsequently.

**Fig. 6:**
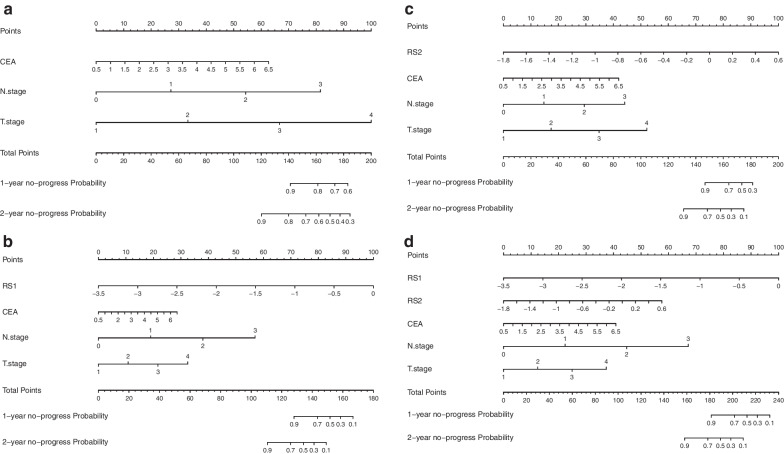
Established clinical nomogram and various clinical-and-radiomics nomograms according to the data before and after treatment. Nomogram **A**, including selected clinical risk factors of Table 2, Nomogram **B**, referring to add RS1 on the basis of clinical risk factors, and Nomogram **C**, referring to add RS2 on the basis of clinical risk factors, for one- and two-year progression-free survival in patients with NPC. Nomogram **D**, that was composite clinical-and-radiomics model, for one- and two-year progression-free survival in NPC patients

Our nomogram integrated different kinds of prognostic and determinant variables, and assigned a corresponding number of points for given variable size, and then, the next step was that the cumulative scores of all variables were matched to the result scale [23] to get possibility of event. Finally, results were displayed in the form of vivid radiomics nomogram. All are shown in the attached Fig. 6.

### Verification of the radiomics nomogram

**Fig. 7:**
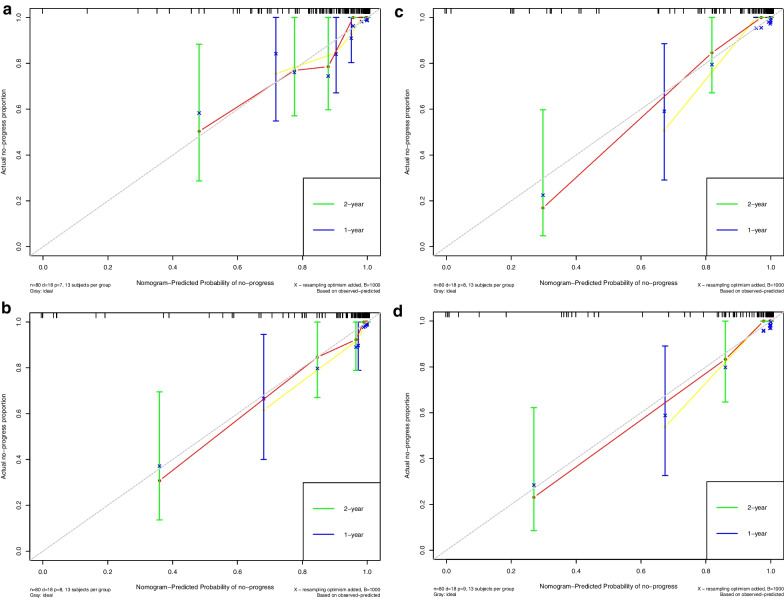

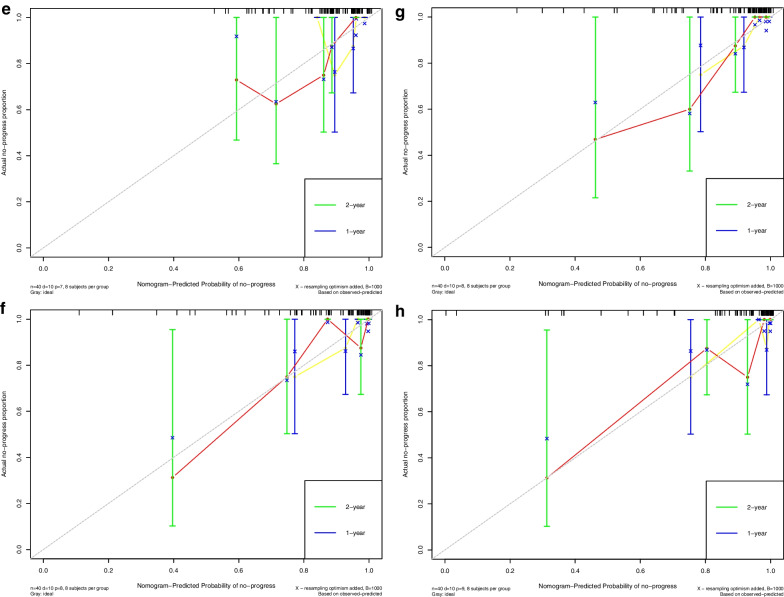
Calibration curves of clinical nomogram and clinical-and-radiomics nomograms in the training (**A**, **B**, **C**, **D**) and validation cohorts (**E**, **F**, **G**, **H**)

Calibration curves (Fig. 7) were generated to compare the consistency between the observed results and the progress predicted by each nomogram. All the results showed good agreements.

Calibration curve indicated the calibration of each model in terms of consonance between the nomogram-evaluated probability of progression-free-survival situation and the observed outcomes of progression-free-survival rate. In the graph of calibration curves, the probability of progression predicted by nomogram was plotted on the *x* axis, while the actual rate was plotted on the *y* axis. The diagonal dotted grey line indicated an ideal model for perfect prediction (the estimated results were completely consistent with the actual results). The yellow and red solid line indicated the performance of nomogram for one-year and two-year, and a closer fit to the dashed line indicated higher accuracy.

**Fig. 8:**
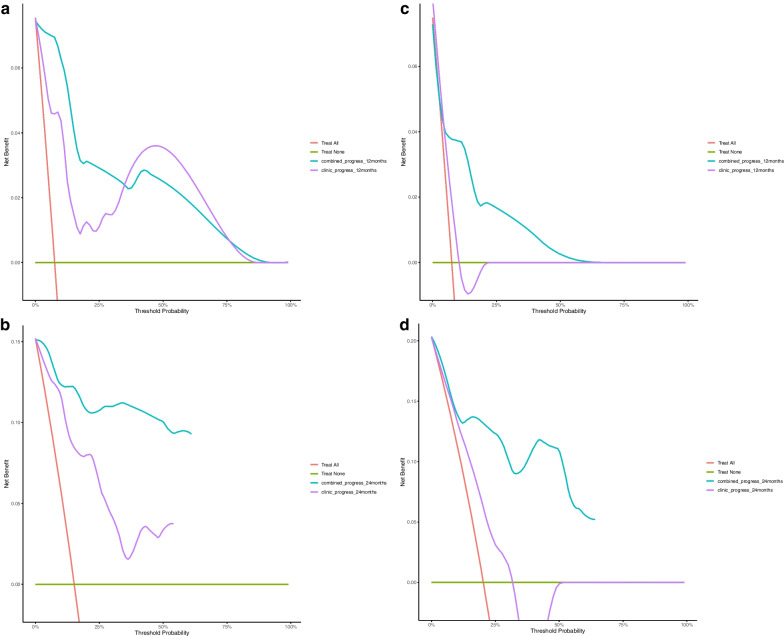
Decision curve analysis for clinical and radiomics nomogram models in the training **A**, **C** and validation cohorts **B**, **D**. The red line represented the net benefit. The x-axis represented high-risk threshold probability. The purple line and blue line represented net benefit of the clinical model and clinical-and-radiomics nomogram, respectively

The decision curve analysis (Fig. 8) showed that the radiomics nomogram combined with clinical risk factors across the range of reasonable threshold probabilities had higher overall net benefits than the simple clinical nomogram.

### Prognostic validation of Rad-score

**Fig. 9:**
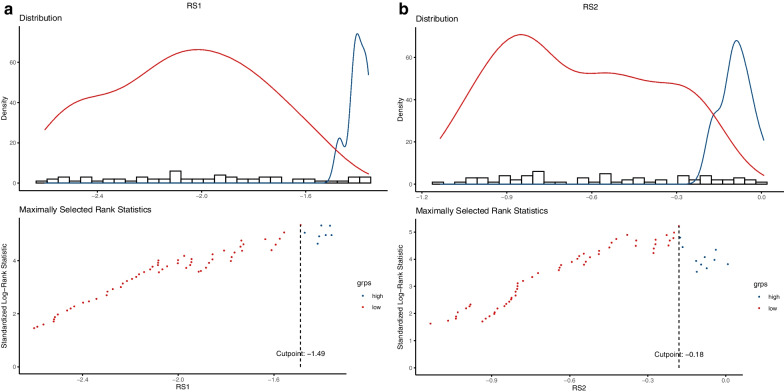
Threshold diagram of Rad-score (RS): **A** RS1. **B** RS2

**Fig. 10:**
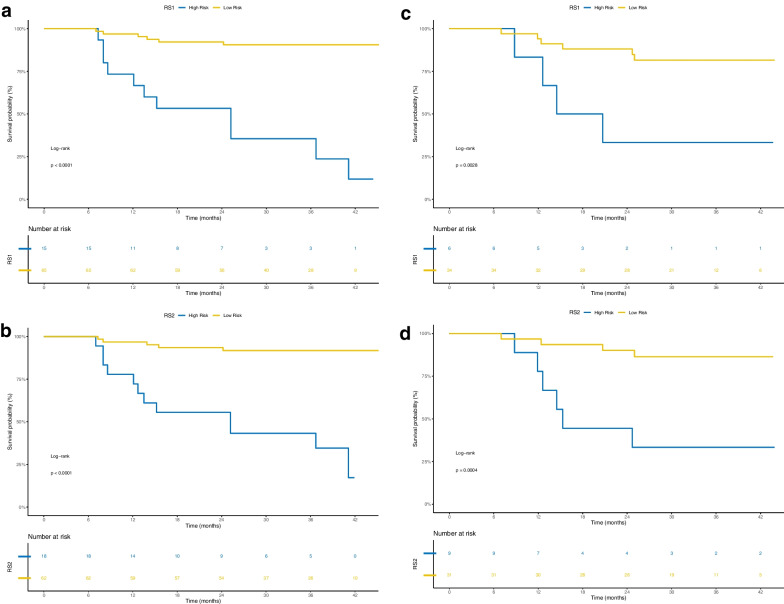
Kaplan–Meier diagrams of NPC patients stratified according to RS1 and RS2, and it displayed a statistically significant difference for PFS with log-rank *P* value < 0.05. The risk table is shown at the bottom of the plots. PFS survival curve of patients in the training cohort: **A** RS1, **B** RS2. PFS survival curve of patients in the validation cohort: **C** RS1, **D** RS2

Rad-score (RS) has been verified to be an independent predictor of prognosis for nasopharyngeal carcinoma patients. The threshold of RS was determined by the Youden index. The value where was corresponding to the maximum Youden index was the optimal cutoff value. Threshold of RS1 (cutoff value, − 1.488) and RS2 (cutoff value, − 0.180) made patients classified into high-risk or low-risk groups (Fig. 9). Logrank test was used to evaluate the significant difference between the survival curves of high-risk and low-risk groups. The results suggested that subtyping by radiomics in two cohorts was striking correlated with survival. Kaplan–Meier analysis was used for survival analysis. In the present study, we set disease progression as the ending event, that is, local recurrence and/or distant metastasis. Potential association between Rad-score and survival was analysed using Kaplan–Meier estimator. The details of statistical analysis are described in the following Fig. 10.

Blue parts represented patients at high risk of disease progression and red parts represented patients at low risk of disease progression. The cutoff value was shown in vertical dashed line.

## Discussion

In this study, we discussed the feasibility of employing an MR imaging-based radiomics signature for progress prediction in II–IVA NPC patients. Radiomics signature when combined with clinical factors containing TNM staging information, showed excellent predictive performance and distinguished from TNM staging alone remarkably.

Fortunately, our research was not censored, which led to fewer deviations. These patients in the study lived in southern China, where nasopharyngeal carcinoma is prevalent. As a high incidence area, we can serve as a crucial example to enrich radiomics results in the field of NPC.

MRI was chosen as our research subject for radiomics analysis of nasopharyngeal carcinoma. This is because that MRI has advantages of superior contrast of soft tissue [24], vivid display of anatomical structure, multiplanar scanning and imaging, and so on. It can display intake of contrast agent clearly and less show radiological artifacts and sclerotic artifacts of bone beam [14]. It can directly determine involvement of parapharyngeal space, and show invasion, extension or displacement of tissue fascia. It’s more effective than CT in showing tumor recurrence and detecting complications of radiotherapy in follow-up of nasopharyngeal carcinoma. Speak something else, in past research, such as the brain research [25, 26], MRI had been extensively and frequently used to depict margin of tumors. Accordingly, even though CT is the most common imaging modality for making treatment plans, MRI is still the first choice for diagnosing, local staging and tumor contouring of nasopharyngeal carcinoma.

In our study, ROIs were the whole tumor, excluding regional lymph node. As for how to sketch ROIs, there was a significant deficiency in the accuracy of automatic segmentation. Therefore, we still regarded researchers' manual segmentation as a ground truth in this study, despite the lack of demarcation standards and the variability among observers [27].

Many factors, such as age, gender, smoking status, the plasma Epstein-Barr virus deoxyribonucleic acid (EBV DNA) status, serum lactate dehydrogenase (LDH) level and high-sensitivity C-reactive protein (hs-CRP) level, had been proved to affect the recurrence of NPC [28–30]. All those had been taken into account when predicting individualized prognosis. Selecting independent factors and then incorporating them with radiomics signatures, the radiomics models were established to predict progression-free survival and tumor recurrence.

Nowadays, the scope of detection and diagnosis has expanded than past decades significantly. Advances in upgrading of imaging machines, improvements in imaging hardware, new imaging protocols, innovations in imaging agents and innovations in mathematical transformation analysis, have made it possible to provide better spatial resolution and allow analysis of tumor heterogeneity [14, 31, 32]. Compared with the past, the role of medical imaging was limited to disease diagnosis, radiomics analysis can be mined by high-throughput feature extraction algorithm [8, 33]. The generated features are used to capture phenotypic differences between tumors. Not constrained in the field of nasopharyngeal carcinoma in effect, radiomics explores its clinical value in various types of tumors by quantitatively measuring macroscopic disease features within and between tumors. What’s more that viewed at the current level of statistical modeling, we are looking forward to using composite models to discover parts that are relevant to treatment response and patients’ prognosis from these features for evidence-based clinical decision support.

Radiomics has been applied to the research of various cancers, for example, head and neck cancer, esophageal cancer, colorectal cancer, lung cancer and breast cancer [20, 22, 34–36]. Previous studies [10, 27, 37–39] have shown good prognostic value of radiomics features, but it is limited to pre-treatment characteristics. Kang et al. [40] established the joint model based on pre-treatment and mid-treatment radiomics signatures, with good prognostic ability, for predicting disease progression or death hazard of locally advanced NPC. Our advantage lies in the fact that extracting not only pre-treatment imaging but also post-treatment imaging for analysis. It is the only one that substantiates the predictive effect of the post-treatment radiomics features on PFS in NPC. We found that the use of radiomics information of pre- and post-treatment can more comprehensively evaluate the therapeutic effect for NPC and improve the prediction efficiency.

This research still has some limitations. First, because the researchers on radiomics are still in its infancy, the amount of available data is relatively small and most of them are retrospective. While collecting and retrieving both MRI and clinical data, there may be selection bias. The retrospective characteristic is an inherent limitation here. Thus, it is necessary for us to conduct further prospective research to confirm the conclusion of this research. Second, this study was conducted in the First Affiliated Hospital of Wenzhou Medical University. Limited by the nature of single institution, we cannot guarantee that results are applicable to patients from other regions and institutions. As a result, it makes sense to proceed large cohort studies in conjunction with other centers for external validation, which can identify the consistent prognostic value and improve the reliability of our study. Furthermore, we only extracted features from ROI representing primary tumor, but did not consider other metastatic lesions including lymph nodes. Analysis of lymph node lesions alone or in combination with primary tumor may have some values, too.

## Conclusions

In conclusion, we constructed a composite clinical-and-radiomics nomogram that integrated independent clinical predictors with pre-treatment and post-treatment radiomics signatures to predict the progression-free survival condition in NPC patients. It may contribute to individualized risk stratification, influence individualized treatment strategies and monitor clinical processes.

## Supplementary Information

**Additional file 1**. Results of time AUC and time C-index about cross-validation of radiomics models.

## Data Availability

The datasets used and analyzed during the current study are available from the corresponding author on reasonable request.

## Abbreviations

NPC: Nasopharyngeal carcinoma
MRI: Magnetic resonance imaging
RS: Rad-score
PFS: Progression-free survival
IMRT: Intensity modulated radiation therapy
TNM: Tumor-node-metastasis
T2WI: T2-weighted image
CE-T1WI: Contrast-enhanced T1-weighted image
AJCC: American joint committee on cancer
UICC: Union for international cancer control
NCCN: National comprehensive cancer network
DICOM: Digital imaging and communications in medicine
PACS: Picture archiving and communication system
TR: Repetition time
TE: Echo time
FOV: Field of view
Mat: Matrix
FA: Fip angle
NEX: Number of excitations
ROI: Region of interest
GLCM: Gray level cooccurrence matrix
GLRLM: Gray level run length matrix
GLSZM: Gray level size zone matrix
GLDM: Gray level dependence matrix
NGTDM: Neighbouring gray tone difference matrix
LASSO: Least absolute shrinkage and selection operator
RFE: Recursive feature elimination
RF: Random forest
mRMR: Minimum-redundancy maximum-relevancy
MI: Mutual information
C-index: Harrell’s concordance index
ROC: Receiver operating characteristic
CI: Confidence interval
WBC: White blood cell
PLT: Platelet
HGB: Hemoglobin
NEUT: Neutrophil
CEA: Carcinoembryonic antigen
EBV DNA: Epstein-barr virus deoxyribonucleic acid
LDH: Lactate dehydrogenase
hs-CRP: High-sensitivity C-reactive protein
