# Supplementary material for: A nomogram model based on pre-treatment and post-treatment MR imaging radiomics signatures: application to predict progression-free survival for nasopharyngeal carcinoma
Source: Radiat Oncol. 2023 Apr 11;18:67. doi: 10.1186/s13014-023-02257-w (PMC10088158; doi:10.1186/s13014-023-02257-w)
Supplement: Supplementary file 1 — Additional file 1. Results of time AUC and time C-index about cross-validation of radiomics models. [file 13014_2023_2257_MOESM1_ESM.docx]

**Supplementary material**

1. Results of cross-validation of the developed clinical and radiomics models using 200 times repeated 5-fold method.
   1. Table S1
   2. Figure S1

1.1 Table S1. Average values of time AUC and time C-index for each model in 200 times repeated 5-fold cross-validation. The differences between every models were significant (*P-value < 0.05).

| Model | 12months_AUC | 24months_AUC | 12months_C-index | 24months_C-index |
| --- | --- | --- | --- | --- |
| CEA+N stage+T stage | 0.573 | 0.704 | 0.573 | 0.678 |
| RS1+CEA+N stage+T stage | 0.800 | 0.840 | 0.791 | 0.815 |
| RS2+CEA+N stage+T stage | 0.652 | 0.777 | 0.648 | 0.746 |
| RS1+RS2+CEA+N stage+T stage | 0.836 | 0.871 | 0.825 | 0.846 |
| P-value | < 0.001* | < 0.001* | < 0.001* | < 0.001* |

1.2 Figure S1. Distribution diagram of time AUC and time C-index of the models in 200 times repeated 5-fold cross-validation.


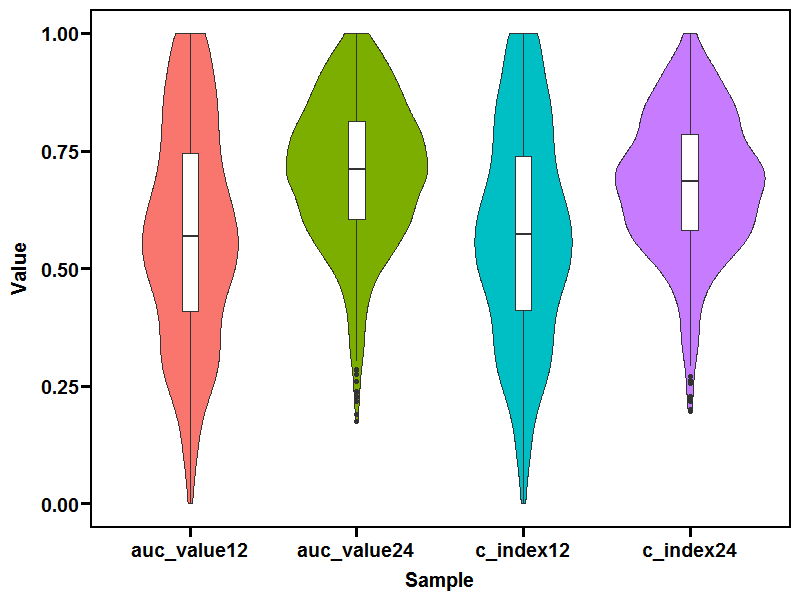

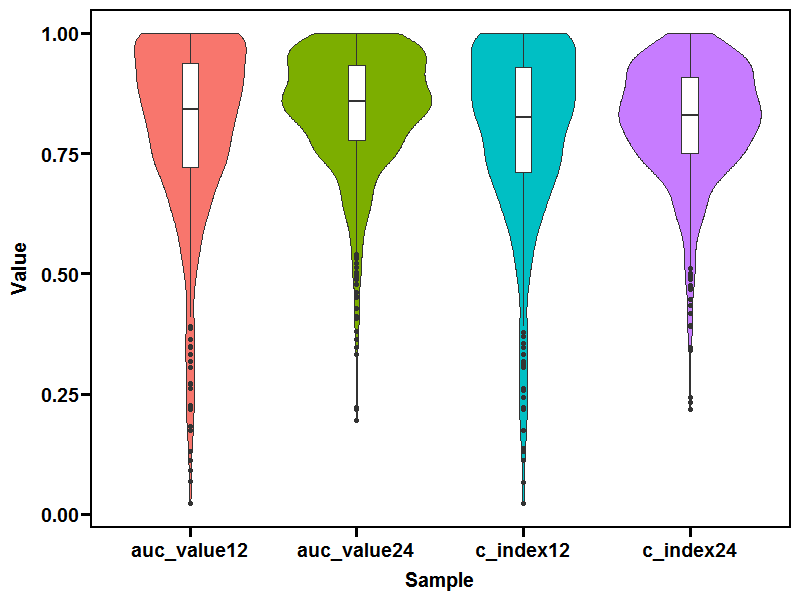
A B


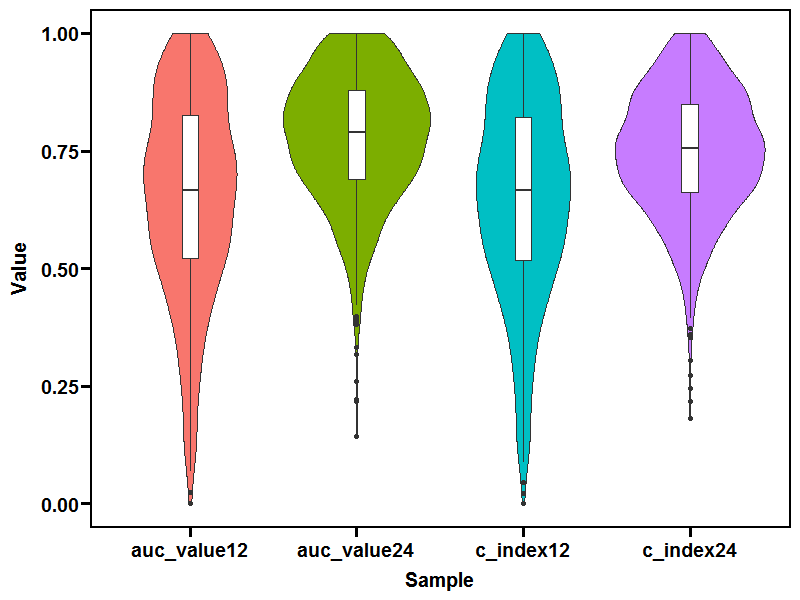

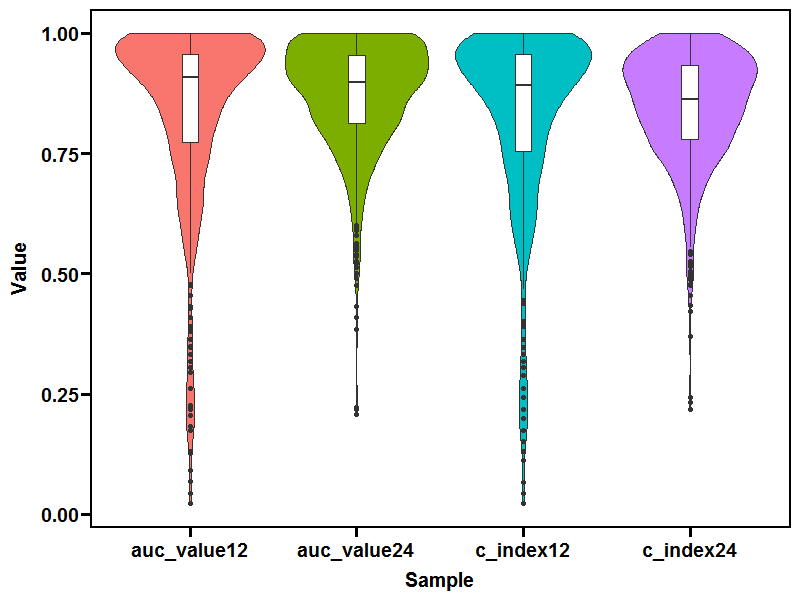
C D
